# Obituary

**Published:** 1855-03

**Authors:** 


### OBITUARY.

Died, at his residence in Philadelphia, Thursday, February 8th, 1855, aged thirty-five years, Samuel Wallace Hall, M.D., a native of Kentucky.

Dr. Hall graduated in New York, at the Crosby Street Medical School, having attended three full courses of lectures. After practising several years in this city, he repaired to Paris, where he remained upwards of two years, prosecuting his studies under Louis, Andral, and other eminent men of the time. On his return to the United States, he resumed his practice with prompt and unusual professional success. He subsequently married a young lady of fortune, the daughter of a Quaker family in Philadelphia, to which city he removed, relinquishing a lucrative practice, and resided there until his premature decease, the immediate cause of which was exposure in attending an aggravated case of typhoid fever; he saved the life of his patient, but lost his own, (as many a noble fellow has done before, and passed unhonored to his forgotten grave,) but such is the peril of medical life.

The distinguishing trait of Dr. Hall, as a physician, was simplicity of prescription, and next to that was the unwavering constancy with which he followed up the case, after he had first settled the diagnosis in his own mind. Within a week of his death he said to me, "What I wanted was a correct diagnosis, but none of them would ever give it to me."

On one occasion, a gentleman of fortune had what one might suppose a very trivial ailment, *a sore toe;* but the pain was such for days and weeks, as to be almost unendurable. Several country physicians had exhausted their skill, without affording even a slight relief. Finally, this gentleman was advised to have the member taken off, in order to save the limb. He concluded, however, that he would first consult Dr. Hall, who at once seeing the nature of the affection, ordered a cotton rag, saturated with sweet oil, to be kept constantly applied ; in a few days, entire relief was procured, and without any return of the ailment for these eight years.

The same resort to safe and simple means, made efficient by his thorough knowledge of the nature of the case, was observable in all his practice. My brother had a great aversion to any thing that had the least appearance of being *unprofes-*

*sional*, of attracting practice by announcements or professions of any description, not even making the offer of his services to the public through a newspaper, on his first arrival at his adopted home. No one would ever have supposed, from the small, plain silver plate of "Dr. Hall" on his door, that the owner had enjoyed, for so many years, all the advantages which New York and Paris could afford, but in all his life, that same plain and unpretending taste was steadily manifested; his idea was, that "true merit will sooner or later find its level, and needs no outside influences." He always felt his power, even to the last moments of his existence, for within an hour of his departure, he wrote an appropriate prescription for himself, in a plain legible hand.

His moral and religious character is to be gathered from his daily life, rather than from any particular act, or from the unreliable and hysterical expressions merely of a dying hour. A man's destiny is decided for eternity, by the mass of his life, through a Saviour's merits.

Dr. Hall's ancestry have been Presbyterian for generations: he was the child of baptism, and prayer, and of the church, to which he was united a number of years ago, under the ministry of Rev. N. L. Rice, D.D., now of St. Louis. He was married in Philadelphia, June 27, 1849, by the Rev. Albert Barnes, D.D. Within a week of his death, hundreds of miles from his family, whom he was extremely anxious to see, and among whom he had expressed the strongest desire to die, when every day might be his last, or any hour, he preferred to lay by, saying, "*It is not well to travel on Sunday.*" It is perhaps difficult to conceive of any circumstances more justifiable of Sunday travelling, yet it weighed nothing with him. He did lay by, and lived three days after his arrival home.

We miss him much; yet we cannot but feel that we shall see him again; the thought ever arises,

> Our loving brother gone before!
> To that unknown and silent shore!
> Shall we not meet, as heretofore,
> Some sunny morning?

What avails him now, the kindly words of the officiating minister, the good and venerable Dr. McKinney; the tasteful habiliments of his mortal body, the comely drab, the coffin of costly black, edged with plain but burnished silver, the long line of carriages, the beautiful place of sepulture in the Cemetery of the Woodlands, on the banks of the Schuylkill, shadowed by the beachlet at his feet, amid marble monuments to others' memory ?—nothing, all!

We shall miss his cordial grasp of welcome, even after short absences, and that subdued and kindly utterance, which nothing could exceed,—"I'm glad to see you!"

We shall miss him in memory of our pedestrian tour to Menai Bridge, the Slate Quarries of Wales; then, through Ireland, to the Giant's Causeway, to the land of Burns, to Alloway Kirk, and the bridge thereby; in our walks through murky London, and cheery Paris; the Strand, the Boulevards; the Champs D'Elysees. We shall miss him in memory too of our breakfast with George Combe, at Edinburgh; of our visit to Morning Side, and its distinguished occupant, the ruddy and merry-faced Chalmers; of Stokes, and Ramedge, and Marshall Hall, and Louis, and Lawrence, and the burly O'Connell in prison: and of our visit to Windsor; the booming of the loud-mouthed cannon; the trampling of mounted troops, as they rode up the long avenue to the Castle; the heralds of Louis Philippe and Prince Albert, who followed in their regal chariot, with its gay outriders, and their foaming steeds: in every cup of all these youthful memories, there will hereafter be a bitter drop, down to life's close. But rest, Brother! rest thee well, with thy little yearling Carrie, who has already followed, and now at thy side sleeps sweetly, as infant innocence only can. It will not be long before we shall hear the summons, too; meanwhile, we'll live in hope of another meeting in the better land, where we shall be always healthy, always happy, and always good.

